# COMMUNITY-BASED SERVICES AND OLDER ADULTS: A COMBINATION IN MENTAL HEALTH PROMOTION

**DOI:** 10.1093/geroni/igz038.3112

**Published:** 2019-11-08

**Authors:** Glaucia Salgado, Sharon Koehn

**Affiliations:** SIMON FRASER UNIVERSITY, Vancouver, British Columbia, Canada

## Abstract

Mental health promotion among older adults has been considered an important goal by the World Health Organization (WHO, 2017). Mental health has been understood as not necessarily the absence of mental illness, but in fact, points on a continuum that although are not mutually exclusive, and it may intersect at times (Cowen, 1991; Keyes & Westerhof, 2012). With such complex health component, medical practices—although extremely critical in many cases—are often one factor of in the equation. Other practices, such as positive relationships among individuals and measures to tackle isolation are relevant and successful when planning practice for mental health promotion (Wister & McPherson 2014; Newall & Menec, 2019). A study done at a community-based seniors service in Vancouver – Canada shows that these spaces are considered a critical resource for visible minority older adults. Two focus groups were conducted at a community-based senior service with visible minority older adults between ages 55 to 80 years old. Results show that visible minority older adults strongly rely on this sector to maintain the connection with the society, and to the services provided in the wider community. Community-based seniors service provide opportunities for social inclusion and interactions, learning new things, and it has an inverse association with feeling isolated and lonely at home—a constant issue stated in the research. These findings indicate the critical role of this sector in ameliorating and promoting the mental health of visible minority older adults.

